# The Bile Acid-Sensitive Ion Channel (BASIC) Is Activated by Alterations of Its Membrane Environment

**DOI:** 10.1371/journal.pone.0111549

**Published:** 2014-10-31

**Authors:** Axel Schmidt, Pia Lenzig, Adrienne Oslender-Bujotzek, Jana Kusch, Susana Dias Lucas, Stefan Gründer, Dominik Wiemuth

**Affiliations:** 1 Institute of Physiology, RWTH Aachen University, Aachen, Germany; 2 Institute of Physiology II, University Hospital Jena, Friedrich-Schiller-University Jena, Jena, Germany; 3 Instituto de Investigação do Medicamento (iMed.ULisboa), Faculdade de Farmácia, Universidade de Lisboa, Lisboa, Portugal; University of Tokyo, Japan

## Abstract

The bile acid-sensitive ion channel (BASIC) is a member of the DEG/ENaC family of ion channels. Channels of this family are characterized by a common structure, their physiological functions and modes of activation, however, are diverse. Rat BASIC is expressed in brain, liver and intestinal tract and activated by bile acids. The physiological function of BASIC and its mechanism of bile acid activation remain a puzzle. Here we addressed the question whether amphiphilic bile acids activate BASIC by directly binding to the channel or indirectly by altering the properties of the surrounding membrane. We show that membrane-active substances other than bile acids also affect the activity of BASIC and that activation by bile acids and other membrane-active substances is non-additive, suggesting that BASIC is sensitive for changes in its membrane environment. Furthermore based on results from chimeras between BASIC and ASIC1a, we show that the extracellular and the transmembrane domains are important for membrane sensitivity.

## Introduction

The bile acid-sensitive ion channel (BASIC) is a member of the DEG/ENaC family of ion channels [1]. DEG/ENaC channels share a common topology, they consist of two transmembrane domains (TMDs) that are linked by a large, glycosylated, extracellular domain (ECD) and cytosolic N- and C-terminal domains [2]. Based on the crystal structure of chicken acid-sensing ion channel (ASIC) 1 it is assumed that DEG/ENaC channels form homo- or heterotrimeric complexes and that the pore region is formed by the TMDs [3]. While the function of many DEG/ENaC channels is known for some time, the physiological role of BASIC is still unknown. It is mainly expressed in hepatocytes and cholangiocytes of the liver, furthermore it is found in the intestinal tract and in the brain [4], [5]. In mouse brain BASIC is restrictively expressed in unipolar brush cells of the vestibulocerebellum [6]. In humans its expression is restricted to the intestinal tract [7].

The electrophysiological properties of BASIC from rat, mouse and human are strikingly different. Rat and human BASIC are strongly inhibited by physiological concentrations of extracellular divalent cations and carry only small non-selective currents at rest [4], [8]. A large variety of stimuli, including membrane stretch and osmotic challenges do not increase the activity of rat BASIC [4]. The low-activity resting state of rat and human BASIC is in contrast to mouse BASIC, which is only partially inhibited by extracellular cations and therefore shows high constitutive activity and high selectivity for Na^+^ [8]. In addition, rat and human BASIC can be activated by the fenamate flufenamic acid (FFA), while mouse BASIC is not affected by FFA. A common feature of the BASIC orthologs, however, is the inhibition by the diarylamidine diminazene (DIMI) [9].

The identification of bile duct-lining cholangiocytes as expression site in the liver led to the identification of bile acids as natural and putatively physiological activators of rat and human BASIC [5]. The mechanism of activation, however remains completely unknown.

Bile acids are amphiphilic molecules with a steroid nucleus, which serve a diverse range of functions. Their classical and probably main task is to aid lipolysis in the intestinal tract by forming lipid micelles allowing an easier access for digestive enzymes. Furthermore they are crucial for maintaining cholesterol homeostasis and recently they have received increasing attention as signaling molecules [10]. Two receptor proteins are known that are directly regulated by bile acids, (i) the nuclear bile acid receptor FXR (farnesoid X receptor) which is involved in controlling bile acid synthesis and export, lipogenesis, gluconeogenesis and several other metabolic processes [11], [12] and (ii) the TGR5 receptor, a G-protein coupled receptor for bile acids residing in the plasma membrane. TGR5 is involved in controlling glucose homeostasis and gallbladder relaxation, and affects inflammatory pathways [13].

Compared to the bile acid receptors FXR and TGR5, relatively high concentrations of bile acids are required for activation of rat and human BASIC [5], [14] and since bile acids are natural detergents, and thus membrane-active molecules, two modes of activation are possible. First, bile acids activate the channel directly by binding to the protein, inducing conformational changes eventually leading to the opening of the channel pore. This would suggest that BASIC is a classical ligand-gated channel and bile acids are its natural ligand. Second, due to their amphiphilic structure, bile acids can interfere with the plasma membrane and alter the channel’s lipid surroundings. The alteration of the membrane properties might then indirectly induce the activation of the channel. This would imply that the channel is not a bile acid-dependent ligand-gated channel but rather a channel that is sensitive to its lipid environment and possibly a sensor for membrane alterations.

In this study we aimed to analyze the mode of activation and combined a pharmacological approach using compounds with known effects on plasma membrane properties with a chimeric approach using channel chimeras derived from BASIC and the related ASIC1a, which, as we show here, is insensitive to bile acids. We identified various membrane-active molecules that affect BASIC activity and we show that various regions of BASIC are important to make it sensitive to bile acids. Collectively, our results suggest that (i) BASIC is sensitive to changes occurring at the level of its surrounding plasma membrane and that (ii) the entire structure of the channel rather than individual subdomains of the channel is crucial for this sensitivity.

## Experimental Procedures

### Molecular biology

Clones for rat BASIC (accession No. NM_022227), mouse BASIC (accession No. NM_021370), rat ASIC1a (accession No. NM_024154) and HyNaC 2, 3 and 5 (accession No. AM393878, AM393880 and FN257513) were described previously [8], [15], [16]. Chimeras between BASIC and ASIC1a were generated by overlap PCR and PCR fragments were cloned into the expression vector pRSSP6009 [15] using the In-Fusion cloning kit (Invitrogen, Germany). Sequences were confirmed by sequencing. Plasmid DNAs were linearized using the restriction endonucleases MluI or NaeI. cRNA was synthesized from linearized plasmids using the SP6 mMessage mMachine kit (Ambion, USA).

### Electrophysiology

Oocytes were surgically removed from tricainemethanesulfonate anaesthesized adult female *Xenopus laevis* and stored in OR-2 medium (in millimolar, 82.5 NaCl, 2.5 KCl, 1.0 Na_2_HPO_4_, 5.0 2-[4-(2-hydroxyethyl)piperazin-1-yl]ethanesulfonic acid (HEPES), 1.0 CaCl_2_, 1.0 MgCl_2_, 0.5 g l^−1^ polyvinylpyrrolidone, pH 7.3). Oocytes (stage V-VI) were injected with 0.8 ng (rASIC1a) or 8 ng cRNA (rBASIC and chimeras) and incubated in low Na^+^ OR-2 medium (in millimolar, 5.0 NaCl, 77.5 N-methyl-d-glucamine, 2.5 KCl, 1.0 Na_2_HPO_4_, 5.0 HEPES, 1.0 CaCl_2_, 1.0 MgCl_2_, 0.5 g l^−1^ polyvinylpyrrolidone, and 10 µM amiloride, pH 7.3) at 19°C.

Two-electrode voltage clamp experiments were performed 24–48 h post-injection as described previously [5], [9]. Briefly, whole-cell currents were recorded in standard bath solution (in millimolar, 140 NaCl, 1.8 CaCl_2_, 1.0 MgCl_2_, and 10 HEPES, pH 7.4) at a holding potential of −70 mV and amplified by a Turbo Tec-03x amplifier (npi electronics, Tamm, Germany). An automated, pump-driven system in combination with an oocyte testing carousel (OTC) (npi electronics, Tamm, Germany) guaranteed full solution exchange within <1 sec [17]. Chemicals were purchased from Sigma or Merck. Fluorescent NBD-derivatized ursodeoxycholic acid (UDCA-NBD) was synthesized as described previously [18].

Confocal patch-clamp fluorometry (PCF) experiments to simultanously monitor rBASIC activation/deactivation and UDCA-binding/unbinding time courses in outside-out patches were performed as described previously [19] 48–72 h post-injection. Current recordings in membrane patches excised from *Xenopus laevis* oocytes were performed using an Axopatch200B amplifier (Molecular Devices, Sunnyvale, CA) and the ISO3 hardware and software (MFK, Niedernhausen, Germany). Sealing and patch excision were realized in low sodium solution (in millimolar, 135 NMDG, 5.0 NaCl, 1.8 CaCl_2_, 1.0 MgCl_2_, and 10 HEPES, pH 7.4), current recordings were realized in normal sodium bath solution (in millimolar, 140 NaCl, 1.8 CaCl_2_, 1.0 MgCl_2_, and 10 HEPES, pH 7.4). The pipette solution contained (in millimolar) 90 K^+^ gluconate, 5.0 NaCl, 2.0 MgCl_2_, and 2.0 EGTA, pH 7.3. The holding potential was −50 mV. Current recording rate was 2 kHz.

To monitor UDCA binding, 50 µM UDCA-NBD [18] was applied together with 1.6 mM unlabeled UDCA. To automatically localize the position of the patch-membrane the bath solution was stained using the red dye DY647 (1 µM, Dyomics, Jena, Germany) [19]. DY647 was applied together with UDCA-NBD. All substances were added to the bath solution. UDCA-NBD concentration jumps (5 sec) were performed using a double-barreled glass pipette (Hilgenberg GmbH, Malsfeld, Germany) mounted on a piezo-driven device producing a parallel laminar solution flow. Confocal images were recorded with an LSM710 confocal microscope (Zeiss, Jena, Germany), which was triggered by the ISO3 software (MFK, Niedernhausen, Germany). A 488 nm Argon laser and a 633 nm He-Ne laser were used to excite UDCA-NBD and DY647, respectively. Imaging recording rate was 10 Hz.

### Data analysis

Two-electrode voltage clamp data were collected and pooled from at least two preparations of oocytes isolated on different days from different animals, if not stated otherwise. Tauroursodeoxycholic acid (UDCA) and other modulatory substances were applied to oocytes for 10 sec. Current amplitudes were determined 10 sec after application, even though in some cases steady-state was not fully reached. Data were analyzed using the Software IgorPro (Wave metrics, USA). Concentration-response curves were fitted using the Hill equation:
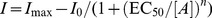
(1)where I_max_ is the maximal current, I_0_ is the residual current in the absence of UDCA, EC_50_ is concentration at which half-maximal responses occur, [A] is the concentration of UDCA, and *n* is the Hill coefficient.

Patch-clamp fluorometry current and binding traces were fitted using either of the two following equations:
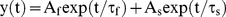
(2a)

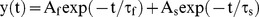
(2b)where τ_f_, τ_s_, A_f_, A_s_ are respective fast and slow time constants and their relative contributions. Fits of equations to data points were performed using the Origin8 software (OriginLab, Northhampton, USA).

Data are reported as mean ± SEM, and statistical significances were evaluated by one-pair ANOVA followed by Tukey’s multiple comparison test using the GraphPad Prism 6.0e software (GraphPad Software, La Jolla, USA).

## Results

### Rat BASIC is modulated by chlorpromazine, trinitrophenol and gadolinium

**Figure 1:**
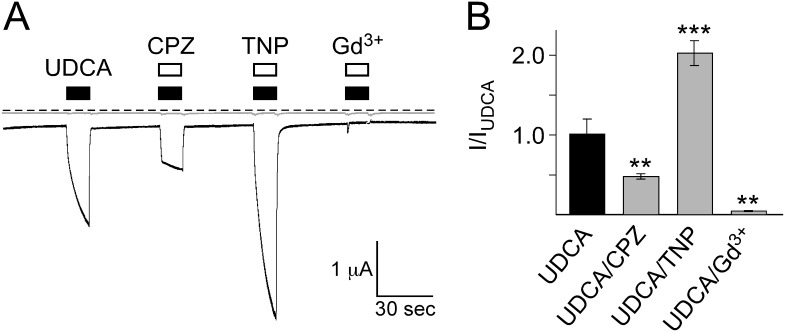
BASIC responses to bile acid are modulated by the membrane active substances chlorpromazine, trinitrophenol and Gd^3+^. A) Representative current traces of an oocyte expressing BASIC (black trace) and a water-injected control oocyte (grey trace). Application of 2 mM UDCA induced a typical BASIC current. Co-application of 0.5 mM CPZ strongly decreased the UDCA-dependent current while co-application of 2 mM TNP strongly increased the current amplitude. The UDCA-dependent current was completely abolished by 100 µM Gd^3+^. Dotted line represents the 0 current level. B) Quantitative comparison of current amplitudes induced by UDCA alone or together with 0.5 mM CPZ, 2 mM TNP or 100 µM Gd^3+^. Currents were normalized to the current induced by UDCA alone, which had an amplitude of 1.1±0.2 µA (*n* = 10). Error bars represent SEM. Statistical significances were tested using one-way ANOVA followed by Tukey's multiple comparison test versus UDCA, **p<0.01, ***p<0.001.

**Figure 2:**
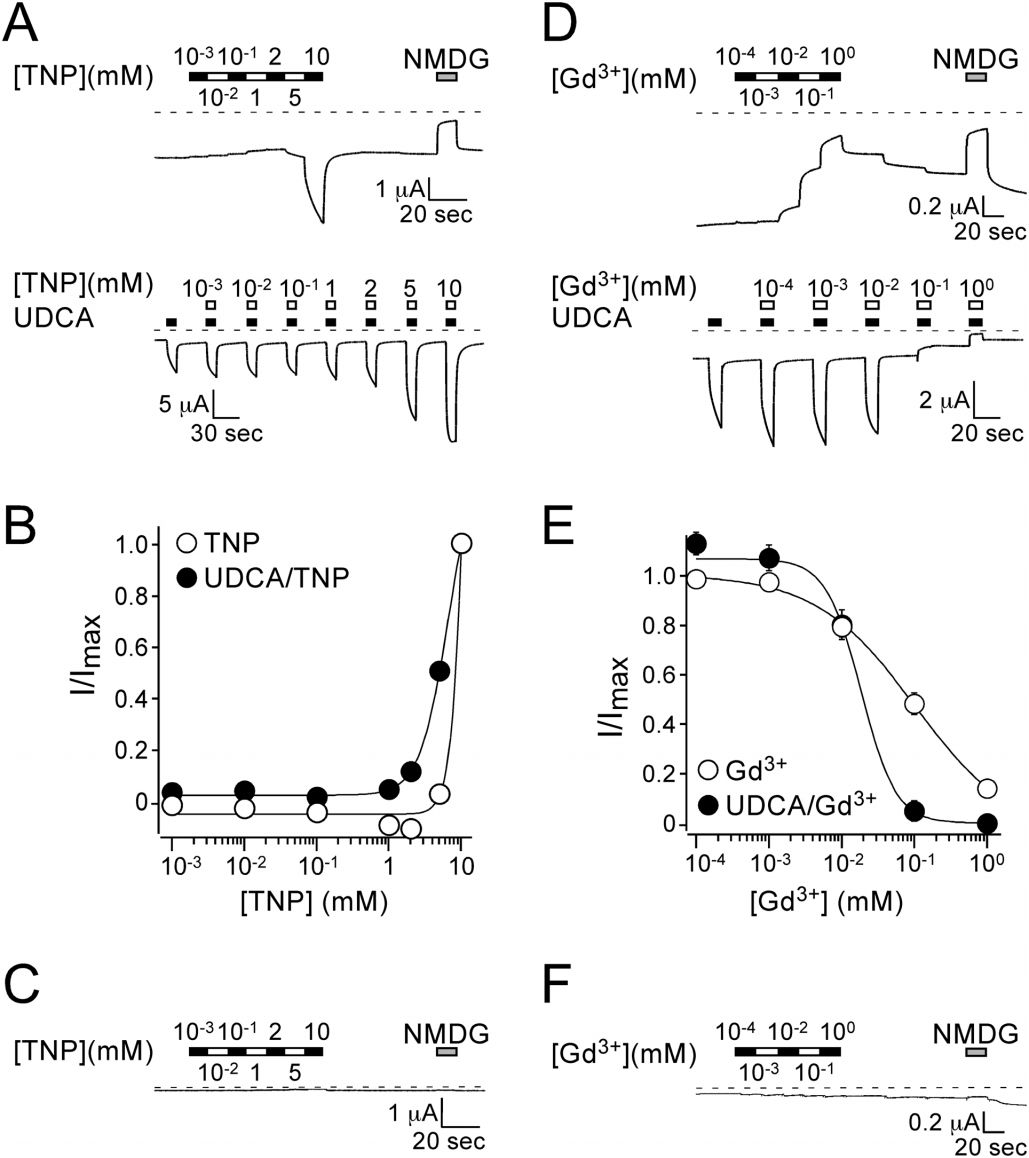
TNP increases while Gd^3+^ decreases BASIC activity in a dose-dependent manner. A) Representative current traces showing the concentration-dependent activation of BASIC by TNP. Upper panel, increasing concentrations of TNP were applied to a rBASIC expressing oocyte. Lower panel, increasing concentrations of TNP were co-applied with 2 mM UDCA. Dotted lines represent the 0 current level. B) Concentration-response curves for TNP in the absence (open circles) and presence (closed circles) of 2 mM UDCA. Currents were normalized to the maximum currents in the presence of TNP, which had amplitudes of 3.9±0.4 µA and 12.1±2.5 µA (n = 10) in the absence and presence of 2 mM UDCA, respectively. The current amplitude of 2 mM UDCA alone was 4.1±0.4 µA (*n* = 10). Error bars represent SEM, curves were fitted to the Hill equation. C) Representative current trace of a non-injected oocyte treated with increasing concentrations of TNP. D) Representative current traces showing the concentration-dependent inhibition of BASIC by Gd^3+^. Upper panel, increasing concentrations of Gd^3+^ were applied to a rBASIC expressing oocyte. Lower panel, increasing concentrations of Gd^3+^ were co-applied with 2 mM UDCA. Dotted lines represent the 0 current level. E) Concentration-response curves for Gd^3+^ in the absence (closed circles) and presence (open circles) of 2 mM UDCA. Currents were normalized to the maximum currents in the absence of Gd^3+^, which had amplitudes of 1.2±0.2 µA and 3.2±0.6 µA (*n* = 10) in the absence and presence of 2 mM UDCA, respectively. Error bars represent SEM, curves were fitted to the Hill equation. F) Representative current trace of a non-injected oocyte treated with increasing concentrations of Gd^3+^.

If bile acids activated rBASIC indirectly via their interaction with the lipid bilayer, we reasoned that other membrane active substances should also affect the activity of the channel. Trinitrophenol (2,3,6 Trinitrophenol, TNP) and the antipsychotic drug chlorpromazine (CPZ) are known pharmacological modulators of the order and shape of lipid bilayers affecting the activity of various ion channels [20]–[25]. The lanthanoid gadolinium (Gd^3+^) is an inhibitor of numerous ion channels, in particular channels that are sensitive to membrane stretch [26], [27]. To test the role of the plasma membrane for bile acid activation of rBASIC we applied TNP, CPZ and Gd^3+^ together with 2 mM of tauroursodeoxycholic acid (UDCA), which is found in rat bile [28] and strongly activates rBASIC (EC_50_: 2.5 mM) [14]. All three substances indeed strongly influenced the UDCA-response of BASIC (Fig. 1A). While 0.5 mM CPZ decreased the response of BASIC to 2 mM UDCA 2.0-fold, 2 mM TNP increased the response approximately 1.8-fold, and 100 µM Gd^3+^ completely abolished the UDCA-dependent activation of BASIC (Fig. 1B). Non-injected oocytes did not respond to the substances (Fig. 1A). Next we determined the dependence of rBASIC activity on the concentrations of TNP and Gd^3+^; CPZ was not soluble above 0.5 mM and was therefore not further considered. We applied increasing concentrations of TNP or Gd^3+^ either alone or together with 2 mM UDCA and analyzed current amplitudes. Interestingly, TNP and Gd^3+^ affected both, the low activity resting state and the UDCA-dependent active state of BASIC in a concentration-dependent manner. When applied alone, concentrations of up to 2 mM TNP weakly inhibited BASIC, but 5 and 10 mM TNP strongly activated BASIC, similar to bile acids (0 mM TNP: 0.7±0.1 µA, 10 mM TNP: 3.9±0.4 µA, n = 10). When applied together with UDCA, TNP strongly increased the UDCA-induced response (2 mM UDCA: 4.1±0.1 µA, 2 mM UDCA/10 mM TNP: 12.1±2.5 µA, n = 10) (Fig. 2A). Due to the limited solubility of TNP, its EC_50_ could not be determined precisely but was estimated to be >5 mM (Fig. 2B). Gd^3+^ strongly inhibited both the low-activity resting state and the UDCA-dependent active state (Fig. 2D); in the presence of UDCA the IC_50_ was 19±7 µM and in its absence 102±34 µM (Fig. 2E) (n = 8). Non-injected control oocytes did not respond to TNP or Gd^3+^ (Fig. 2C, F). Taken together these data show that alteration of the membrane can either increase or decrease the bile acid-dependent activation of BASIC, in agreement with a membrane-dependent mechanism of bile acid activation of BASIC.

### Membrane-binding of UDCA correlates with UDCA-activation of rBASIC

Next we studied membrane-binding of UDCA and UDCA-activation of rBASIC using confocal patch-clamp fluorometry [29], a technique which has been successfully used to study the interaction between ligands and channel proteins, e.g. cyclic-nucleotide gated channels [19], [30] and hyperpolarization-activated and cyclic nucleotide-modulated (HCN) channels [31], [32]. Currents were recorded from membrane patches excised from rBASIC expressing oocytes and uninjected control oocytes and binding of fluorescent UDCA-NBD was monitored in parallel by confocal microscopy [19]. Outside-out patches were subjected to fast application of a solution containing 1.6 mM UDCA and 50 µM fluorescent UDCA-NBD [18].

**Figure 3:**
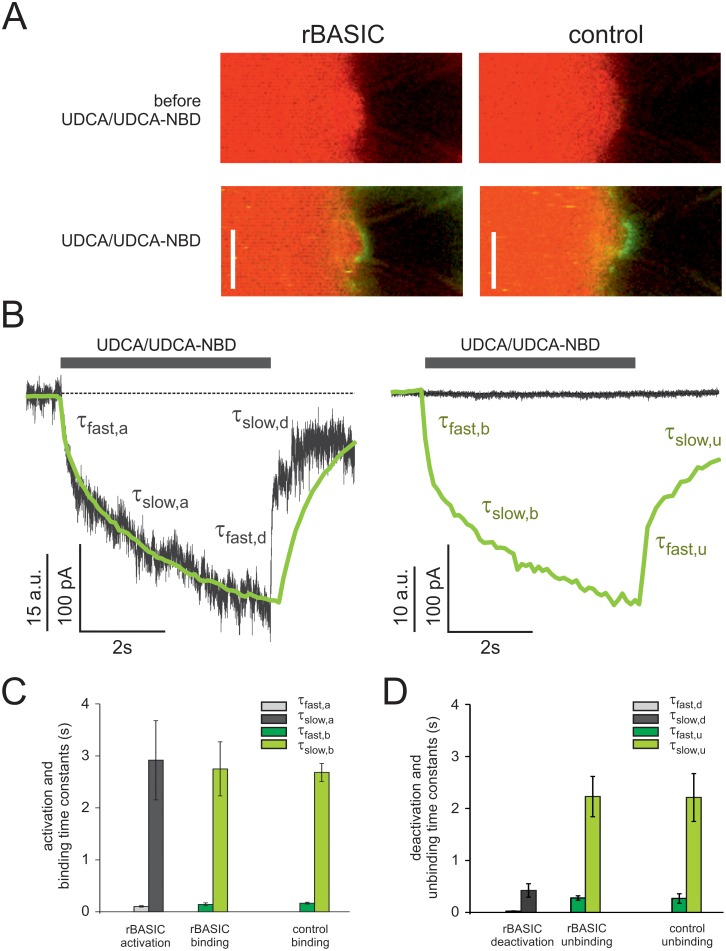
Correlation of UDCA membrane binding and rBASIC activation. A) Confocal images of patch pipettes containing outside-out patches excised from *Xenopus laevis* oocytes. Left panel, membrane patch excised from an oocyte expressing rBASIC, before (upper panel) and after (lower panel) application of UDCA-NBD/UDCA (50 µM/1.6 mM). Right panel, membrane patch excised from an uninjected control oocyte, before (upper panel) and after (lower panel) application of UDCA-NBD/UDCA. Green fluorescence signals originated from UDCA-NBD binding to the membrane, red fluorescence signal originated from DY647 background dye (1 µM) staining the bath solution. The transmission channel is overlayed. Scale bar = 5 µm. B) Binding time courses and current time courses after sudden UDCA-NBD/UDCA concentration jumps, obtained from a membrane patch excised from an rBASIC expressing oocyte (left panel) and a membrane patch excised from an uninjected control oocyte (right panel). Green traces represent fluorescence signals induced by UDCA-NBD binding and unbinding to the patch membranes (for clarity, increase in fluorescence is depicted as downward deflection). Grey traces represent simultaneously recorded current traces. Averages of three consecutive measurements are shown. Time courses of activation/binding and deactivation/unbinding were fitted by the sum of two exponentials (equations (2a) and (2b)). τ_fast_, τ_slow_ represent fast and slow time constants for rBASIC activation/inactivation and UDCA-NBD binding/unbinding events (a, rBASIC activation; b, UDCA-NBD binding; d, rBASIC deactivation; u, UDCA-NBD unbinding). C) and D) Quantitative comparison of slow and fast time constants of rBASIC activation and UDCA-NBD binding (C) and rBASIC deactivation and UDCA-NBD unbinding (D). Error bars represent SEM; *n* = 3–7.

In rBASIC containing membrane patches, fast application of UDCA/UDCA-NBD for 5 sec induced a current increase (161±84 pA, n = 5) (Fig. 3B), similar to UDCA-induced currents in whole oocytes. The maximum current was reached within 3–5 sec, and upon washout of UDCA/UDCA-NBD currents returned to baseline within less than 1 sec. In control oocytes no current was induced by UDCA/UDCA-NBD application (Fig. 3B). The application of UDCA/UDCA-NBD also increased fluorescence from 0.93±0.29 a.u. to 30.4±5.6 a.u. (n = 7) in the dome region of the outside-out patch, which is the area not attached to the glass surface of the patch pipette (Fig. 3A and B). This strong fluorescence increase monitors binding of UDCA-NBD either directly to rBASIC or to the membrane or to both. Upon washout of UDCA/UDCA-NBD the fluorescence decreased again to the pre-application level. Importantly, in membrane patches excised from uninjected control oocytes application of UDCA/UDCA-NBD also induced an increase in membrane-associated fluorescence (from 0.57±0.14 a.u. to 38.1±8.5 a.u., n = 4), similar to the increase in rBASIC containing membrane patches (Fig. 3A and B). This shows that binding of UDCA is independent of the presence of rBASIC in the membrane. Importantly, the application of NBD alone (50 µM NBD-Cl and 1.6 mM UDCA) did only induce a small increase in fluorescence (from 0.33±0.07 a.u. to 1.51±0.1 a.u., n = 3), compared to UDCA-NBD application, indicating that the membrane-associated fluorescence of UDCA-NBD is mainly mediated by the UDCA portion of the molecule.

We compared the kinetics of rBASIC activation with the kinetics of fluorescence increase. The activation of rBASIC can be separated into a fast and a slow phase (Fig. 3B). The time constant τ_fast,a_ for the fast activation phase was 0.10±0.01 s, and the time constant τ_slow,a_ for the slow activation phase was 2.92±0.76 s (n = 5). The time course of the fluorescence increase matched precisely the time course of current activation phase. It also consisted of a fast and a slow component and the corresponding time constants τ (τ_fast,b_: 0.14±0.01 s; τ_slow,a_: 2.75±0.05 s, n = 7), were not significantly different from the time constant of the current time course. The time constants of the fluorescence increase in membrane patches from uninjected control oocytes (τ_fast,b_ 0.17±0.02 s; τ_slow,b_: 2.68±0.02 s, n = 3) did also not differ significantly from the time constants of rBASIC containing membrane patches (Fig. 3C). The perfectly superimposing time courses of current and fluorescence increases suggest that the activation of rBASIC follows the binding of UDCA to the membrane. In contrast, deactivation of rBASIC and the decrease of membrane-associated fluorescence upon washout of UDCA/UDCA-NBD showed different kinetics. Deactivation was faster than the fluorescence decrease (deactivation, τ_fast,d_: 0.024±0.0045 s; τ_slow,d_: 0.422±0.129 s; fluorescence decrease, τ_fast,u_: 0.277±0.044 s; τ_slow,u_: 2.23±0.39 s, n = 4) (Fig. 3D), which could be due a slower washout of the UDCA-NBD compared to UDCA, which is mainly responsible for the current increase.

Taken together these results support the hypothesis that rBASIC activation by UDCA is not due to direct binding of UDCA to the channel but rather due to its binding to the membrane.

### Rat BASIC is activated by various structurally unrelated membrane active substances

**Figure 4:**
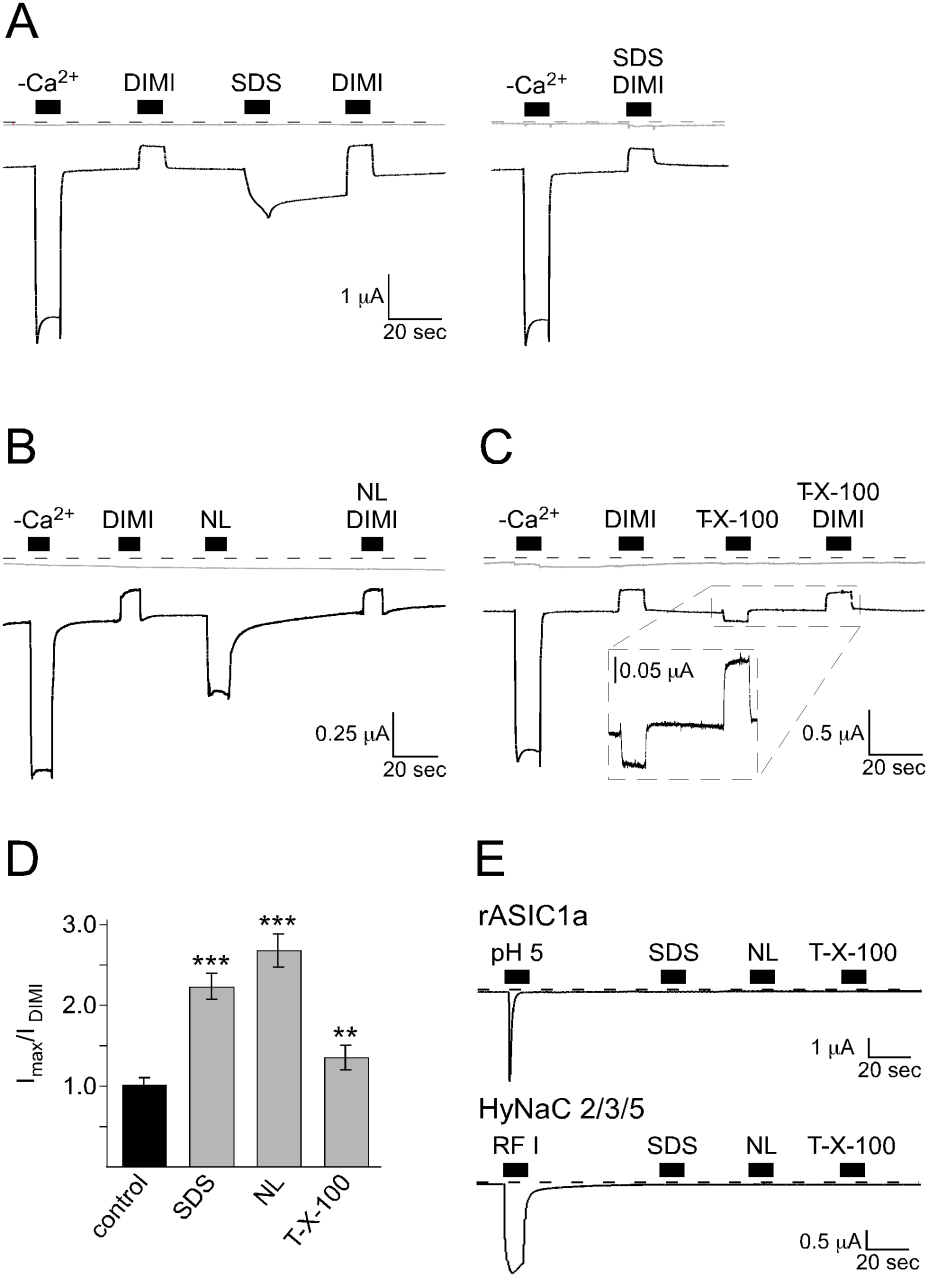
Other membrane-active substances, which are structurally unrelated to bile acids also activate BASIC. A–C) Representative current traces (black) of BASIC-expressing oocytes showing that the application of 400 µM SDS (A, left panel), 500 µM N-Lauroylsarcosine (NL) (B) and 100 µM Triton-X 100 (C) increased the activity of BASIC. The application of 100 µM diminazene (DIMI) alone or together with SDS (A, right panel), NL (B) or Triton-X 100 (C) inhibited BASIC currents. Non-injected oocytes did not respond to the application of the substances (grey traces). Dotted lines represent the 0 current level. (D) Quantitative comparison of current amplitudes induced by SDS, NL or Triton-X 100. Currents were normalized to the DIMI-sensitive current in the absence of stimulatory substances, which had an amplitude of 0.25±0.03 µA (*n* = 8). Error bars represent SEM. Statistical significances were tested using one-way ANOVA followed by Tukey's multiple comparison test versus control, **p<0.01, ***p<0.001, *n* = 8. (E) Representative current traces of oocytes expressing ASIC1a or HyNaC2/3/5. Application of pH 5 or 5 µM RFamide I, respectively, activated the channels, while application of 400 µM SDS, 500 µM NL or 100 µM Triton-X 100 did not.

To further address the question of membrane-dependent activation of BASIC we tested whether other detergent molecules that are structurally unrelated to bile acids also activate BASIC. We included the common anionic detergents SDS and N-lauroylsarcosine (NL) and the nonionic detergent Triton-X 100 in our study. First, we determined sub-solubilizing concentrations of these substances by electrophysiological control measurements with un-injected oocytes. At concentrations of 400 µM (SDS), 500 µM (N-lauroylsarcosine) and 100 µM (Triton-X 100), respectively, the substances did not induce significant current changes (control, 36±1.2 nA; SDS 34±0.6 nA; NL 35±0.8 nA; Triton-X 100 35±0.8 nA, p<0.4, n = 7), indicating that significant permeabilization and solubilization of the plasma membrane did not occur at these concentrations (Fig. 4, grey current traces). In contrast, they induced robust currents in oocytes expressing BASIC. Amplitudes of BASIC currents in the low-activity state were estimated by block with 200 µM diminazene, a blocker of BASIC [9]. Amplitudes of BASIC currents in the high-activity state were estimated by removing extracellular divalent cations (–Ca^2+^), which inhibit the channel at physiological concentrations [8]. The application of SDS induced a 2-fold increase of the low-activity currents (Fig. 4A and D). Upon washout of SDS the current decreased again within seconds but remained at an elevated level, compared to the pre-application level. Only within several minutes the current returned to the pre-application state. This long-lasting current was blocked by diminazene (Fig. 4A), suggesting that it was due to a sustained activation of BASIC. When SDS was applied in the presence of diminazene, it did not increase currents (Fig. 4A). Similar to SDS, NL increased the activity of BASIC, however more potently (3-fold current increase) (Fig. 4B and D). Upon washout of NL the current immediately returned to pre-applications level. When co-applied with diminazene, NL did not increase currents, suggesting it activated BASIC. Triton-X 100 also increased BASIC activity (Fig. 4C and D), however less potently (1.5-fold) than SDS, NL or UDCA. The Triton-X 100 induced current increase was also inhibited by diminazene, supporting that it was mediated by BASIC. The responses of BASIC to NL and Triton-X 100 (Fig. 4B and C) had faster kinetics than the responses to UDCA, TNP and SDS (Figs. 1A, 2A and 4A), which may be due to different membrane partitioning behavior of the substances. Importantly, ASIC1a and the related Hydra Na^+^ Channel (HyNaC) 2/3/5 [16], [33], were not activated by SDS, NL or Triton-X 100 (Fig. 4E).

**Figure 5:**
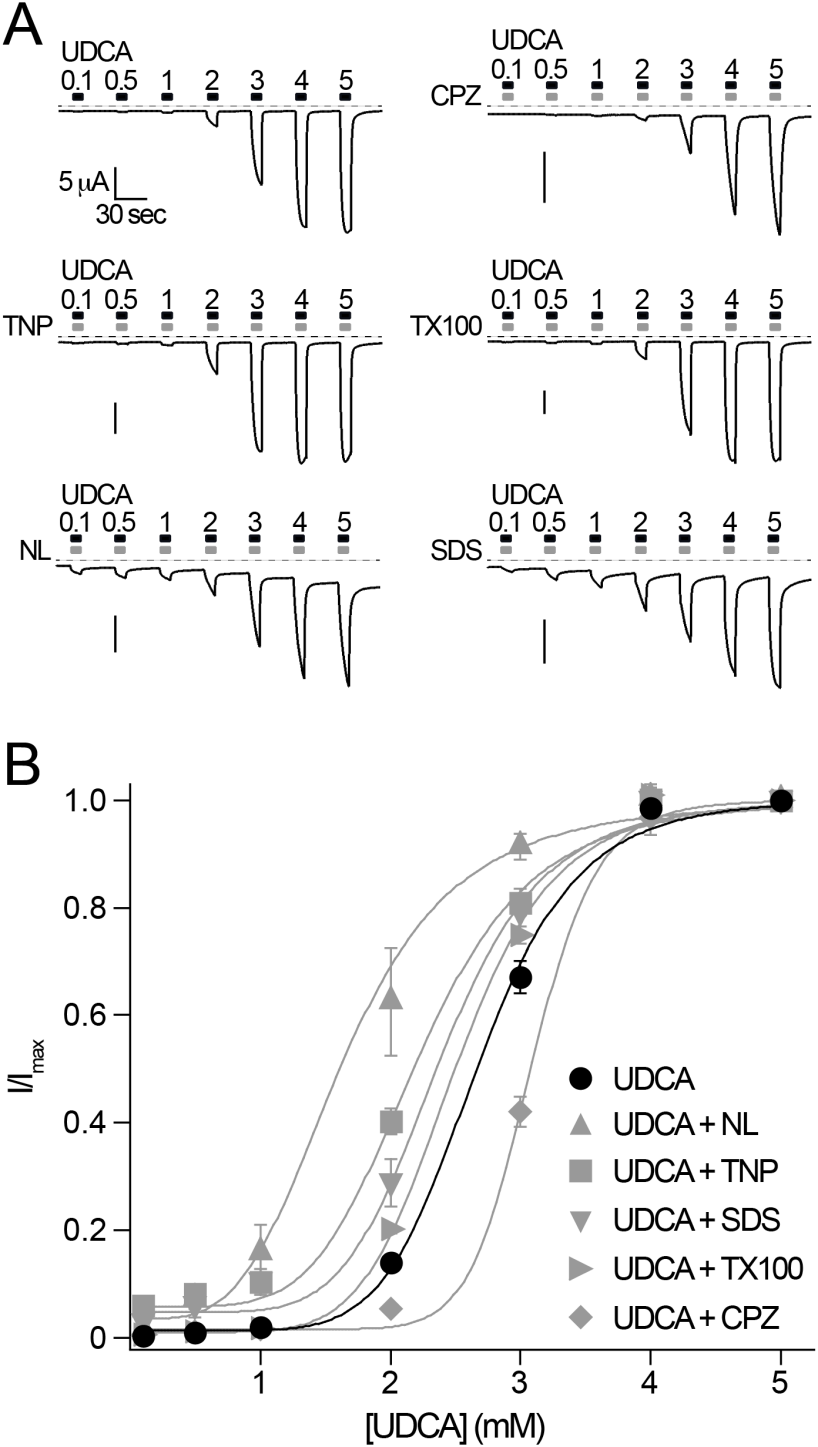
Bile acids and other membrane-active substances activate BASIC by a similar mechanism. A) Representative current traces of BASIC showing the concentration-dependent activation of BASIC by UDCA in the absence and presence of membrane active substances (CPZ, 500 µM; TNP, 2 mM; TX100, 100 µM; NL, 500 µM or SDS, 400 µM). Dotted lines represent the 0 current level B) Concentration-response curves for UDCA in the presence and absence of membrane active substances. Currents were normalized to the maximum current at 5 mM UDCA, which was 23.8±2.4 µA. Error bars = S.E.M., *n* = 10. Curves represent fits to the Hill equation (equation (1)).

To verify whether bile acids and other membrane active substances influence BASIC by a similar membrane-dependent mechanism, we reasoned that the membrane active substances would affect the concentration dependent activation of BASIC by UDCA. Therefore we first determined the concentration dependence of UDCA alone (Fig. 5A and B). The EC_50_ was 2.7±0.06 mM, which is similar as previously reported [14]. Next we performed experiments where the membrane active substances CPZ, TNP, Triton-X 100, NL or SDS were co-applied at constant concentrations with increasing concentrations of UDCA. The presence of the inhibitory substance CPZ indeed shifted the concentration response curve to a significantly higher value (EC_50_ = 3.1±0.03 mM, p<0.001, n = 9). In contrast the presence of the activating substances TNP, T-X-100, NL and SDS shifted the concentration response curves to significantly lower values (Fig. 5A and B) (EC_50_ TNP, 2.2±0.04 mM, p<0.001; EC_50_ Triton-X 100, 2.5±0.01 mM, p<0.05; EC_50_ NL, 1.6±0.07 mM, p<0.001; EC_50_ SDS, 2.4±0.05 mM, p<0.001; n = 8–10), suggesting the same mode of activation of these substances and bile acids. Taken together, these results support the conclusion that BASIC is sensitive for its membrane environment and that bile acids activate BASIC by altering its membrane environment.

**Figure 6:**
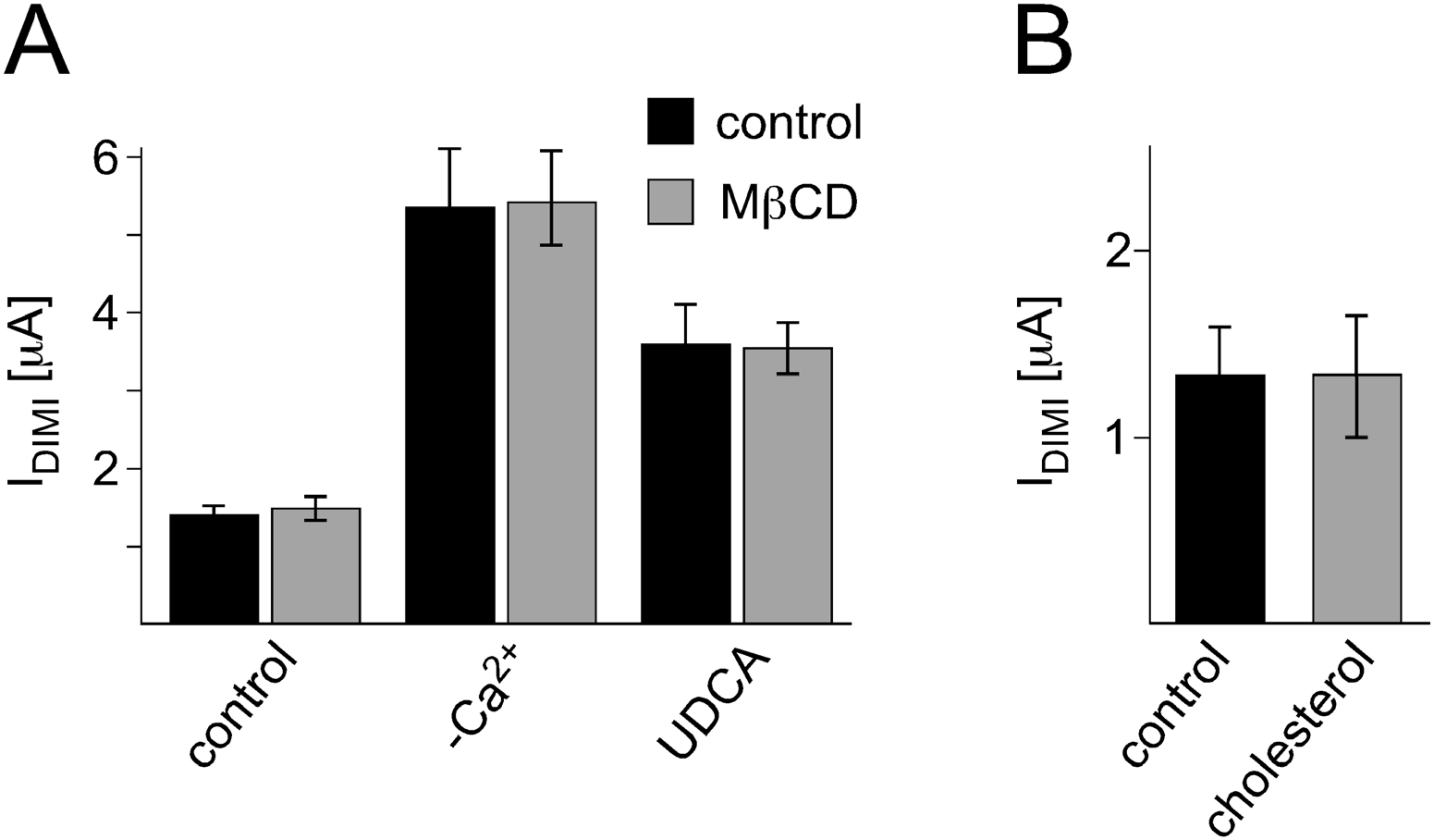
Cholesterol does not affect rBASIC activity. A) Removal of cholesterol does not influence rBASIC activity. Quantitative comparison of DIMI-sensitive rBASIC currents from oocytes that were incubated in low Na^+^ OR-2 medium containing 20 mM MβCD (grey bars) or 20 mM mannitol (control, black bars) for approximately 2 h prior to recordings. Current amplitudes in the absence of any stimulus (control), in the absence of extracellular Ca^2+^ (−Ca^2+^) and in the presence of 2 mM UDCA are shown. B) Addition of cholesterol does not influence rBASIC activity. Quantitative comparison of DIMI-sensitive rBASIC current amplitudes before (black bar) and after 10 sec application of 2 mM MβCD-balanced water-soluble cholesterol (grey bar). Error bars represent SEM, *n* = 8.

Cholesterol is a major constituent of biological membranes and co-determines their properties like membrane fluidity and subdomain organization. Therefore we addressed the question whether the cholesterol content of the membrane affects the activity of rBASIC. Removal of cholesterol from the oocyte membrane can be achieved by incubation with 20 mM MβCD [34]. rBASIC currents were recorded from oocytes incubated in MβCD and control oocytes incubated in 20 mM mannitol. Interestingly, neither the low activity state of rBASIC nor the active state induced by removal of extracellular Ca^2+^ or application of 2 mM UDCA was altered by cholesterol depletion (Fig. 6A). The application of cholesterol to rBASIC expressing oocytes did not affect the rBASIC dependent current either (Fig. 6B). This suggests that cholesterol-dependent membrane properties do not affect rBASIC activity.

### Mouse BASIC is also affected by membrane active substances

**Figure 7:**
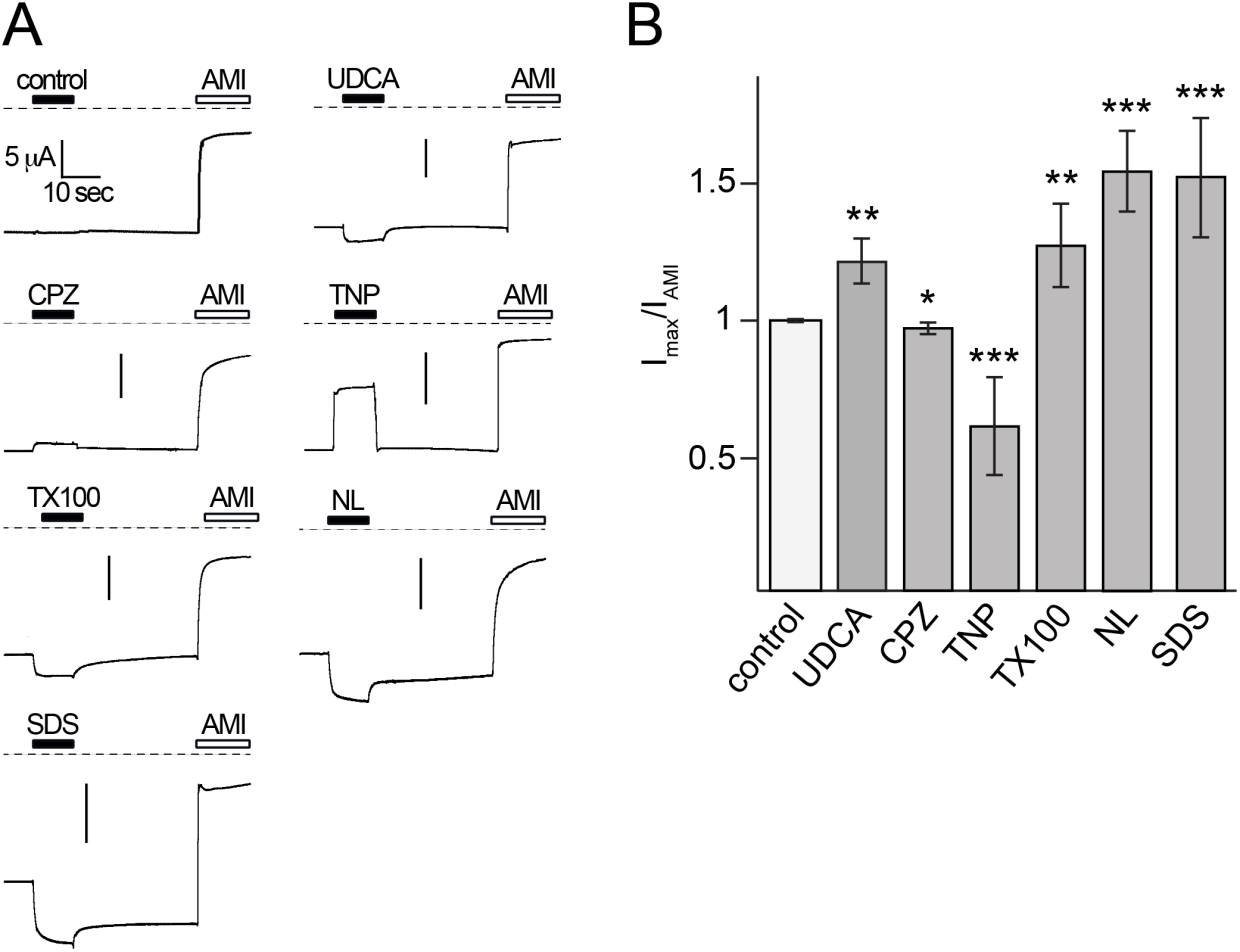
Mouse BASIC is also affected by membrane active substances. A) Representative current traces of mBASIC-expressing oocytes showing that the application of 2 mM UDCA, 500 µM CPZ, 10 mM TNP, 100 µM Triton-X 100, 500 µM NL or 400 µM SDS change the activity of mBASIC. Dotted lines represent the 0 current level. To evaluate the amiloride-sensitive current 100 µM amiloride (AMI) was applied after wash-out of the membrane active substances. (B) Quantitative comparison of current amplitudes induced by UDCA, CPZ, TNP, Triton-X 100, NL or SDS. Currents were normalized to the amiloride-sensitive current, which had an amplitude of 12.3±2.4 µA (*n* = 56). Error bars represent SEM. Statistical significances were tested using one-way ANOVA followed by Tukey's multiple comparison test versus control, *p<0.05, **p<0.01, ***p<0.001, *n* = 8.

In contrast to rat BASIC the ortholog from mouse shows high constitutive activity and strong selectivity for Na^+^. To test whether it is also sensitive to its membrane environment we analyzed the influence of the membrane active substances on the activity of mouse BASIC. We applied UDCA, CPZ, TNP, Triton-X 100, NL and SDS at the same concentrations as previously tested for rat BASIC to oocytes expressing mouse BASIC. Mouse BASIC showed amiloride-sensitive currents in the range of 5 to 15 µA (12.3±2.4 µA, n = 56) as previously reported [8] (Fig. 7A). Similar to rat BASIC, the application of UDCA, Triton-X 100, NL and SDS further increased the activity of mouse BASIC 1.2 to 1.5-fold (UDCA, 1.3-fold; Triton-X 100, 1.2-fold; NL and SDS, 1.5-fold) while CPZ decreased the activity of mouse BASIC (0.95-fold) (Fig. 7A and B). Interestingly TNP, which has an activating effect on rat BASIC strongly decreased mouse BASIC activity (0.6-fold), (Fig. 7A and B) suggesting that besides its effect on the membrane it may also block mouse BASIC. These results suggest that the constitutively active mouse BASIC is also sensitive for changes of its membrane environment.

### Structural determinants of bile acid sensitivity

**Figure 8:**
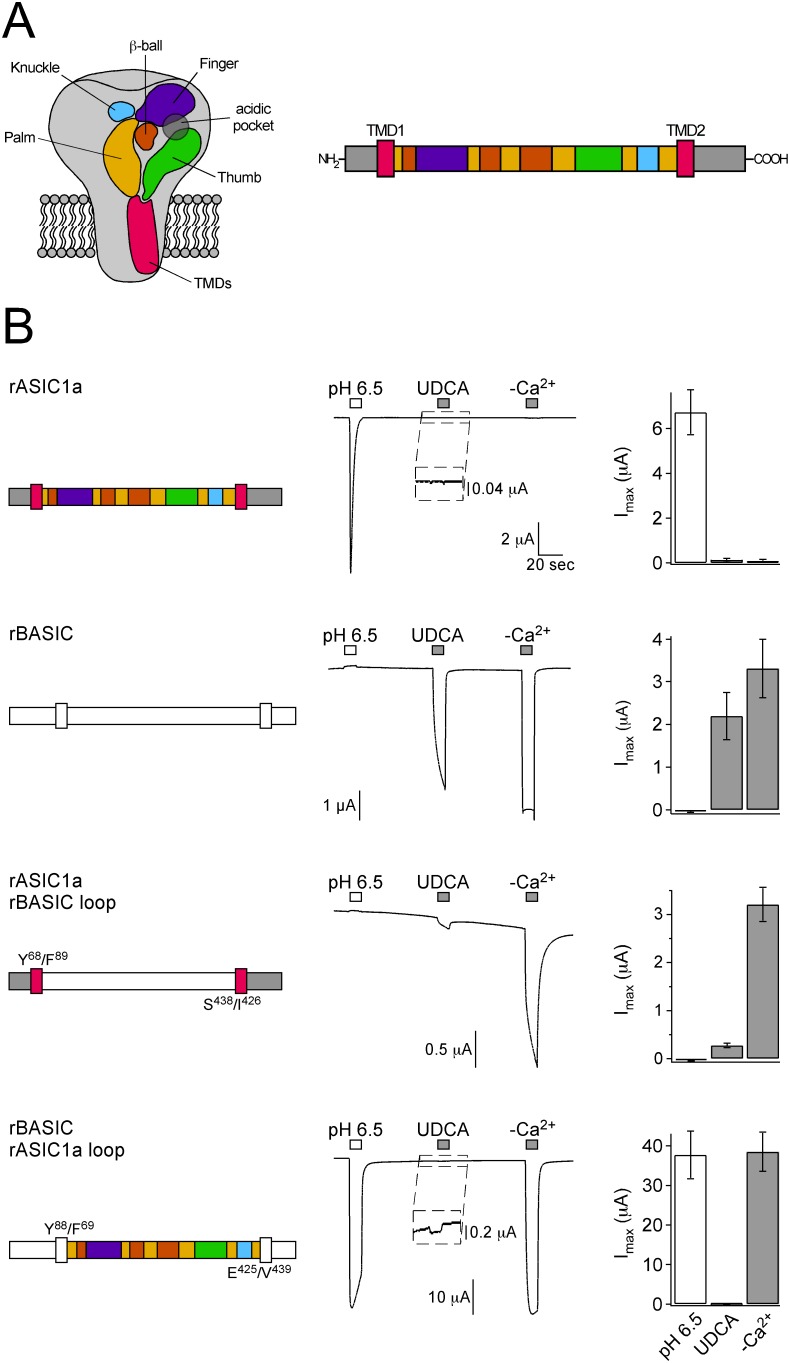
The ECD of rBASIC is involved in bile acid sensitivity. A) Left, scheme of ASIC1 showing the domain organization of one subunit as suggested by Jasti *et al.* based on the crystal structure of chicken ASIC1 [3]. The ECD consists of five domains: palm (yellow), thumb (green), finger (violet), knuckle (blue) and β-ball (orange). Right, linear scheme of ASIC1a showing the domains in the same color coding as in the scheme on the left. Presumably, BASIC shows a similar domain organization as ASIC1a. B) Left panel, schematic drawings of rASIC1a, rBASIC and chimeras; rASIC1a is depicted as in A), for clear distinction, rBASIC is depicted in white. Residues defining the borders of the rASIC1a and rBASIC sequences are shown. Middle panel, representative traces of currents induced by the subsequent application of pH 6.5 and 2 mM UDCA followed by the removal of divalent cations (–Ca^2+^). Right panel, quantitative comparison of current amplitudes induced by pH 6.5, UDCA and divalent cation removal. Error bars represent SEM; *n* = 6–12. Note that different amounts of cRNA were injected (rASIC1a, 0.8 ng; rBASIC, rASIC1a-rBASIC-loop and rBASIC-rASIC1a-loop, 8 ng, respectively).

Next we addressed the question which structural features of BASIC are important for its bile acid-sensitivity. To achieve this we intended to generate chimeras between BASIC and a related channel, which is insensitive to bile acids. Therefore we tested whether rat ASIC1a, which is activated by a sudden increase in extracellular protons, is insensitive to bile acids. Application of 2 mM UDCA did indeed not activate ASIC1a (Fig. 8B). In contrast, application of pH 6.5 activated ASIC1a but not BASIC, and application of 2 mM UDCA or removal of divalent cations (–Ca^2+^) strongly activated BASIC but not ASIC1a (Fig. 8B). Like previously reported [8], BASIC was weakly inhibited by pH 6.5. We exploited these findings and constructed chimeras between ASIC1a and BASIC. We exchanged various regions of these channels, including the TMDs and the ECD or portions of it. Transitions between sequences of ASIC1a and BASIC were positioned in regions that do not form α−helices or β-sheets in the cASIC1a structure [3]. We then tested activation of the chimeras subsequently by low pH, the bile acid UDCA, and removal of divalent cations. This application protocol allows the identification of domains, which either confer bile acid-sensitivity to ASIC1a, or disrupt bile acid-sensitivity of BASIC.

Since a classical extracellular ligand most likely binds to the large ECD of BASIC, we first exchanged the complete ECDs of BASIC and ASIC1a (“loop” chimeras). Fusion of the ECD of BASIC to the TMDs of ASIC1a (chimera rASIC1a-rBASIC-loop) rendered the channel weakly sensitive to UDCA. Even though the activation of this chimera by UDCA was weak compared to wild-type BASIC, it suggests that the ECD is indeed involved in bile acid sensitivity of BASIC. Interestingly, in the complementary experiment, in which the ECD of ASIC1a was transferred to the TMDs of BASIC (rBASIC-rASIC1a-loop), UDCA-sensitivity was also observed, however, compared to the response to removal of divalent cations the response to UDCA was extremely weak (Fig. 8B).

**Figure 9:**
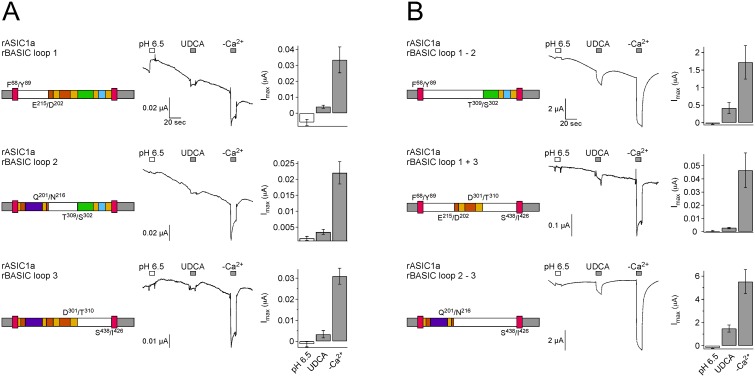
Several regions of the ECD of rBASIC are involved in bile acid sensitivity. A, B) Left panel, schematic drawings of chimeras; rASIC1a portions are depicted as in Fig. 5A, rBASIC portions are depicted in white. Residues defining the borders of the ASIC1a and BASIC sequences are shown. Middle panel, representative traces of currents induced by the subsequent application of pH 6.5 and 2 mM UDCA followed by the removal of divalent cations (–Ca^2+^). Right panel, quantitative comparison of current amplitudes induced by pH 6.5, UDCA and divalents removal. Error bars represent SEM; *n* = 6–12.

To narrow down the region within the ECD involved in bile acid sensitivity and to potentially identify a bile acid-binding pocket, we divided the ECD of BASIC into three portions of similar length and exchanged them for the corresponding regions of ASIC1a (chimeras “rASIC1a-rBASIC-loop 1”, “-2” and “-3”). Chimeras rASIC1a-rBASIC-loop 1, - 2 and -3 did only generate small currents. Removal of divalent cations, however, consistently induced responses and all three chimeras also responded weakly to UDCA (Fig. 9A), suggesting that the bile acid sensitivity is not exclusively associated with either of these short extracellular regions alone. One possible reason for the small currents of the chimeras might be an impaired folding and expression. Therefore we generated chimeras which combined either the first and second (rASIC1a rBASIC loop 1–2), the first and third (rASIC1a rBASIC loop 1+3) or the second and third portion (rASIC1a rBASIC loop 2–3) of the ECD of BASIC. Chimeras “loop 1–2” and “loop 2–3” indeed robustly expressed in oocytes and showed an activation pattern similar to rBASIC: removal of extracellular cations strongly increased the current amplitudes and pH 6.5 weakly reduced them. Importantly, chimeras were also weakly activated by UDCA (Fig. 9B). In summary, all chimeras that involved the ECD, whether they exchanged it entirely or small portions of it, were, compared to activation by removal of divalent cations, similarly weakly activated by UDCA. If the ECD contained a classical ligand binding pocket, however, we would have expected that some chimeras would not have been activated by UDCA at all, or that chimeras had at least significant differences in UDCA-sensitivity, depending on whether the binding pocket was included or not. Therefore, we conclude from this first set of chimeras that the ECD of BASIC contributes to its bile acid-sensitivity but found no evidence for a classical ligand binding site in the ECD.

We then explored the role of the TMDs. TMDs could either bind bile acids or mediate sensitivity to the membrane surroundings. An example for bile acid-binding by TMDs is the bile acid receptor TGR5, for which a recent study using a homology model and docking experiments suggests that bile acids bind to the TMDs [35], while an example for the sensitivity of TMDs to membrane surroundings is the mechanosensitive ion channel MscL from *E. coli* [36].

**Figure 10:**
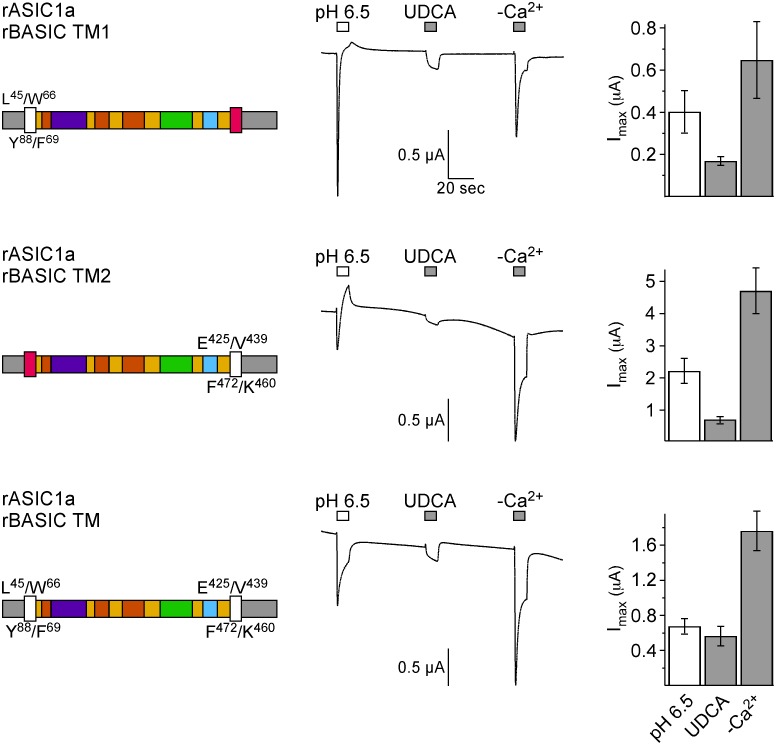
The TMDs of rBASIC are involved in bile acid sensitivity. Left panel, schematic drawings of chimeras; rASIC1a portions are depicted as in Fig. 5A, rBASIC portions are depicted in white. Residues defining the borders of the ASIC1a and BASIC sequences are shown. Middle panel, representative traces of currents induced by the subsequent application of pH 6.5, 2 mM UDCA, and removal of divalent cations (–Ca^2+^). Right panel, quantitative comparison of current amplitudes induced by pH 6.5, UDCA and divalent cation removal. Error bars represent SEM; *n* = 6–12.

DEG/ENaC channels contain two TMDs per subunit and we introduced TMDs 1 and 2 from BASIC either individually or together into ASIC1a (chimeras rASIC1a-rBASIC-TM1, -TM2 or -TM) and then applied the activation protocol as above. These chimeras showed desensitization upon activation by Ca^2+^-removal (Fig. 10), similar to some chimeras, which contained parts of the extracellular loop of BASIC (rASIC1a rBASIC loop 1, loop 2, loop 3, and loop 1+3). This is in contrast to wild-type BASIC, which shows no desensitization upon Ca^2+^-removal and suggests that structural components of ASIC1a confer this property.

Interestingly, all three chimeras were activated by UDCA, chimera 2, which contains TMD2, showed the strongest activation (Fig. 10). However, in contrast to wild-type BASIC but similar to the chimeras involving the ECD, non of the three chimeras involving the TMDs was activated as strongly by UDCA as by removal of divalent cations, indicating that the TMDs are important for bile acid sensitivity but that they are not sufficient for a full response. Again, these results are unexpected if the TMDs contained a bile acid binding site. Collectively, the results from the chimeras revealed no bile acid binding site and rather suggest that different regions of BASIC confer sensitivity to its membrane surroundings.

## Discussion

In this paper we provide pharmacological data suggesting that the bile acid-sensitive ion channel (BASIC) is sensitive to changes in its membrane environment. More specifically, our results indicate that also bile acids, which are potent natural agonists of BASIC, activate BASIC indirectly by changing the membrane properties. Evidence for this conclusion is several-fold. First, different structurally unrelated membrane-active molecules mimic the activation by bile acids of BASIC (Figs. 1, 2A–C, 4). Second, activation of BASIC by bile acids is prevented by some membrane-active substances like CPZ and Gd^3+^ (Fig. 1, 2D–F). Third, UDCA binds to the membrane independently of the presence of rBASIC, and kinetics of membrane binding and BASIC activation are very similar (Fig. 3). Fourth, bile acids and other membrane active substances shift the apparent affinity of rBASIC for UDCA (Fig. 5), suggesting that they activate BASIC by a similar mechanism. Finally, when constructing chimeras between rBASIC and ASIC1a we found no evidence for a bona fide bile acid binding site of BASIC. Our results rather suggest that different regions of BASIC, including the ECD and the TMDs, confer bile acid-sensitivity to BASIC. These results are in full agreement with the idea that bile acids activate BASIC indirectly by changing its membrane surroundings.

### Which property of its membrane surrounding is sensed by BASIC?

The plasma membrane has several properties, which could be sensed by BASIC. Our results suggest that BASIC senses the membrane curvature. The asymmetrical lipid composition of the two layers of the plasma membrane can be responsible for changes in shape that cells undergo upon incubation with amphiphilic substances such as CPZ or TNP [23]–[25]. The negatively charged TNP accumulates in the more positively charged outer leaflet of the bilayer increasing the surface area, inducing positive curvature and thus membrane protrusions, a process termed membrane crenation. In contrast, the positively charged CPZ accumulates in the inner leaflet inducing negative membrane curvature and invaginations of the plasma membrane, termed cup-formation. These changes in membrane curvature might control BASIC activity. According to this hypothesis and the results presented here, the positive curvature induced by TNP would activate the channel, while the negative curvature induced by CPZ would inhibit it.

Bile acids are negatively charged amphiphilic molecules. In erythrocytes, bile acids induce positive membrane curvature [23], similar to TNP. The activating effect of bile acids might therefore be mediated by inducing positive membrane curvature. Co-application of CPZ and UDCA leads to a decrease in current amplitude which is in line with the hypothesis that the direction of the membrane curvature - either positive or negative - is responsible for the activity of BASIC: UDCA induces positive curvature activating BASIC, co-application of CPZ counteracts positive curvature and inhibits BASIC activity. In summary, all results are in agreement with BASIC sensing the membrane curvature. But to unequivocally verify this hypothesis additional experiments using membranes of defined composition and properties would be required.

Besides curvature, the membrane has other properties that could, in principle, be sensed by BASIC. One is membrane stretch. Several ion channels that are modulated by amphiphilic molecules, for example TRPA1, the K^+^ channel TRAAK, and the bacterial channel MscL, are mechanosensitive and also activated by lateral membrane stretching [20], [22], [37], [38]. This is in strong contrast to BASIC, which is sensitive to amphiphilic substances but insensitive to membrane stretching [4]. We conclude that the alterations of the membrane induced by bile acids and other membrane active substances are different from lateral membrane stretching.

Another membrane property is surface charge. The adsorption of charged amphiphilic molecules to the membrane could alter its surface charges that are determined by the charged head groups of the lipids. These charge changes could serve as stimulus for conformational changes of BASIC and its activation or inhibition and thus represent another possible mechanism underlying the activation of BASIC. However, the stimulatory effect of the uncharged Triton-X 100 does not support the possibility that the surface charge plays a crucial role for BASIC activity.

Finally, membrane subdomains might play a role. Several examples are known where the activity of ion channels is dependent upon their localization in membrane subdomains (for review see [39]). The large conductance Ca^2+^-activated K^+^ (BK) channel for example is activated by translocation from cholesterol rich raft domains to non-raft domains [40]. Membrane active substances can influence membrane subdomains. For example, several bile acids including deoxycholic and chenodeoxycholic acid were shown to induce alterations of membrane cholesterol and caveolin, a marker protein for lipid rafts [41], [42]. However, our observation that cholesterol depletion or addition does not influence rBASIC activity, speaks against a major role of membrane microdomains. Nevertheless, further studies are required to elucidate the possible role of membrane microdomains for BASIC activity.

### What is the structural basis of membrane sensitivity of BASIC?

How can BASIC sense changes in its membrane environment? Sensitivity to many structurally unrelated membrane active substances suggests that there is no specific binding site for these substances in the BASIC protein. Moreover, any portion of BASIC transplanted onto ASIC1a, whether derived from the ECD or the TMDs conferred bile acid sensitivity to ASIC1a. However, no portion conferred full sensitivity. These results suggest that BASIC has no bona fide bile acid binding site and that a certain bile acid sensitivity is mediated by different regions of BASIC. In addition, if we consider that rBASIC, in contrast to ASIC1a, has a low activity at rest [4], [8] we propose that the closed state of rBASIC is structurally unstable and that perturbations of its membrane surroundings further destabilize the closed state shifting the equilibrium distribution towards the open state. According to this hypothesis, chimeras containing portions of rBASIC in the structural context of ASIC1a would destabilize the closed state of ASIC1a, thereby conferring a weak bile acid-sensitivity to ASIC1a.

This scenario is in agreement with bile acid-sensitivity of the related ENaC [43]. In *Xenopus* oocytes, there are two populations of ENaC: one with a high open probability P_o_ and one that is nearly silent [44]. Interestingly, bile acids further increase P_o_ of already active channels but do not activate silent channels [43]. This is in agreement with the idea that the population of ENaC with an energetically unstable closed state (high P_o_) senses bile acids and thus membrane alterations whereas the population with a stable closed state (low P_o_) does not. Similarly, ASIC1a has a stable closed state with no activity at rest and is insensitive to bile acids.

Alterations of the membrane can only directly affect the TMDs of BASIC, which are in direct contact with the lipid bilayer. The ECD, however, is also important for membrane sensitivity (Fig. 8). How does the ECD of BASIC influence its membrane-dependent gating?

For ASIC1a it is assumed that proton binding induces long-range conformational changes of the ECD that are transduced to the TMDs via a ball-and-socket like joint structure just above the TMDs [3]. The ASIC1a ECD transplanted onto the TMDs of BASIC renders the chimera almost completely insensitive for its membrane environment (Fig. 8) suggesting that it hinders conformational changes of the TMDs necessary for channel opening. Therefore we speculate that the coupling of the TMDs and the ECD in BASIC is different from ASIC1a. Furthermore it is also possible that the ECD of BASIC has a higher degree of structural flexibility compared to the ECD of ASIC1a, which would allow the movements and opening of the TMDs as a result of membrane changes, whereas the ECD of ASIC1a would not allow TMD movements and channel opening following membrane alterations.

### What is the physiological role of BASIC?

The physiological role of BASIC remains a puzzle, despite the recent progress made on this channel [5], [8], [9], [14], [45]. It is expressed in a variety of organs and tissues including those with a prominent epithelium like the intestinal tract, the liver, the kidney and the lung but it is also found in the brain [4], [5]. Bile acids could represent the physiological stimulus in the liver and the intestinal tract and activate the channel by temporarily altering the membrane microenvironment. However, in the brain, where BASIC is also expressed, bile acid concentrations are too low to activate the channel [5]. But if sensitivity of the membrane surrounding is a crucial feature of BASIC it is possible that the activity of the channel depends on the lipid composition of the cell type the channel is expressed in. The lipid composition of neurons for example could render the channel active in the absence of any stimulus, allowing a weak constant Na^+^ influx and a continuous depolarization of the cell, while in epithelial cells the channel could be inactive and thus dependent on a certain stimulus.

### Outlook

In summary we show that BASIC is sensitive to its membrane environment and suggest that bile acids activate the channel by changing membrane curvature. Whether membrane sensitivity helps BASIC to serve its physiological function remains to be seen.
